# P321: Current status of pharmacovigilance in Africa

**DOI:** 10.1186/2047-2994-2-S1-P321

**Published:** 2013-06-20

**Authors:** S Skalli, H Sefiani, R Soulaymani, R Ouled Errkhis, R Benkirane

**Affiliations:** 1 Anti Poison Center and Pharmacovigilance, Rabat, Morocco; 2 Faculty of Sciences, IbnTofail University, Kenitra, Morocco; 3 Faculty of Medecine and Pharmacy , Rabat, Morocco

## Introduction

Pharmacovigilance systems missions are to ensure medicine safety through efficient and timely collection, assessment, and communication of risks and benefits to support decision making at various levels of the health care system in countries.

InAfrica, the need for pharmacovigilance is increasingly being recognized. In several countries of this continent, public health programs supported by WHO and other partners have introduced pharmacovigilance for antimalarial drugs in these countries. Though, this should be expanded to safety monitoring of all drugs. Pharmacovigilance requires substantial infrastructure, expertise and funding, which are often lacking in African countries.

## Objectives

To describe the current pharmacovigilance in African countries and the contributing factors to its development, Moroccan Pharmacovigilance Center as WHO collaborating center has conducted a study using a questionnaire distributed to all African countries.To analyze data, countries were classified in groups.

## Results

From this study, itappears that there is a good progress in all African countries in the last decade with a difference between the North Africa and the sub-Saharan Africa. Pharmacovigilance development is correlated with the country development, the country language, the health systems development, and the pharmaceutical industry development.

The concept and principles of pharmacovigilance still need time to be fully understood.

## Conclusion

In this matter, leadership and dedicated personnel is essential, advocacy and continuity are a sine qua non condition for pharmacovigilance viability, minimum established staff is needed, linkages with international network are essential, need for pharmacovigilance to be recognized by public health programs and regulators, government and international support is needed and networking with international groups should continue.

## Disclosure of interest

None declared

